# Machine learning for detecting Wilson's disease by amplitude of low-frequency fluctuation

**DOI:** 10.1016/j.heliyon.2023.e18087

**Published:** 2023-07-07

**Authors:** Bing Zhang, Jingjing Peng, Hong Chen, Wenbin Hu

**Affiliations:** a Graduate School of Anhui University of Chinese Medicine,230012, China; b Affiliated Hospital of Institute of Neurology, Anhui University of Chinese Medicine,230031, China

**Keywords:** Wilson's disease, Resting-state functional magnetic resonance imaging, Amplitude of low-frequency fluctuations, Machine learning

## Abstract

Wilson's disease (WD) is a genetic disorder with the A7P7B gene mutations. It is difficult to diagnose in clinic. The purpose of this study was to confirm whether amplitude of low-frequency fluctuations (ALFF) is one of the potential biomarkers for the diagnosis of WD. The study enrolled 30 healthy controls (HCs) and 37 WD patients (WDs) to obtain their resting-state functional magnetic resonance imaging (rs-fMRI) data. ALFF was obtained through preprocessing of the rs-fMRI data. To distinguish between patients with WDs and HCs, four clusters with abnormal ALFF-z values were identified through between-group comparisons. Based on these clusters, three machine learning models were developed, including Random Forest (RF), Support Vector Machine (SVM), and Logistic Regression (LR). Abnormal ALFF z-values were also combined with volume information, clinical variables, and imaging features to develop machine learning models. There were 4 clusters where the ALFF z-values of the WDs were significantly higher than that of the HCs. Cluster1 was in the cerebellar region, Cluster2 was in the left caudate nucleus, Cluster3 was in the bilateral thalamus, and Cluster4 was in the right caudate nucleus. In the training set and test set, the models trained with Cluster2, Cluster3, and Cluster4 achieved area of curve (AUC) greater than 0.80. In the Delong test, only the AUC values of models trained with Cluster4 exhibited statistical significance. The AUC values of the Logit model (P = 0.04) and RF model (P = 0.04) were significantly higher than those of the SVM model. In the test set, the LR model and RF model trained with Cluster3 had high specificity, sensitivity, and accuracy. By conducting the Delong test, we discovered that there was no statistically significant inter-group difference in AUC values between the model that integrates multi-modal information and the model before fusion. The LR models trained with multimodal information and Cluster 4, as well as the LR and RF models trained with multimodal information and Cluster 3, have demonstrated high accuracy, specificity, and sensitivity. Overall, these findings suggest that using ALFF based on the thalamus or caudate nucleus as markers can effectively differentiate between WDs and HCs. The fusion of multimodal information did not significantly improve the classification performance of the models before fusion.

## Abbreviation Full term

ALFF: Amplitude of low-frequency fluctuations
BOLD: Blood oxygen level-dependent
FA: Fractional anisotropy
fMRI: Functional magnetic resonance imaging
FOV: Field of view
GRF: Gaussian random field
HCs: Healthy controls
LR: Logistic regression
MPRAGE: Magnetization-prepared rapid gradient-echo
RF: Random forest
ROC: Receiver operating characteristic
rs-fMRI: Resting-state functional magnetic resonance imaging
SVM: Support vector machine
TE: Echo time
TIV: Total intracranial volume
TR: Repetition time
ROIs: Regions of interest
UWDRS: Unified Wilson's disease rating scale
WD: Wilson's disease
WDs: Patients with Wilson's disease

## 1 Introduction

Wilson's disease (WD) is a genetic disease caused by mutation of the ATP7B gene, which leads to disordered copper metabolism. Worldwide, the prevalence of WD ranges from 1/10,000 to 1/30,000 [1]. In an epidemiological survey of some areas in Anhui Province, the incidence and prevalence of WD were approximately 1.96/100,000 and 5.87/100,000, respectively [2]. Copper accumulation primarily occurs in the liver and brain, as well as in other organs due to the loss of copper transport function by ATP7B [3]. More than half of the WD patients (WDs) suffer from damage to their nervous system and exhibit symptoms such as motor dysfunction, dysarthria, gait abnormality, and dystonia [4,5]. Brain MRI reveals abnormalities in various regions, including the basal ganglia, white matter, midbrain, pons, cerebellum, and thalamus of WDs [[6], [7], [8], [9], [10]]. Compared to healthy controls (HCs), WDs exhibit cortical and subcortical atrophy in the globus pallidus, caudate nucleus, thalamus, frontal lobe, and parietal lobe [11]. However, diagnosing WD correctly is often challenging, leading to treatment delays. If left untreated, copper overload can cause irreversible damage to WDs. Therefore, early diagnosis and standardized treatment are crucial in the management of WD [12].

Resting-state functional magnetic resonance imaging (rs-fMRI) is a noninvasive and stable tool for detecting brain activity and connectivity [13]. Recently, there has been a growing trend of rs-fMRI studies on WD. Tinaz et al.'s [14] study showed that, compared to the control group, non-brain type WDs exhibited significantly reduced functional connectivity between the frontal lobe and striatum, while neurological WDs showed significantly increased functional connectivity between the hippocampi. Additionally, Zhu et al. [15] found that connectivity between the cerebellum and thalamus increased, while connectivity between the cortex and thalamus, cortex and striatum, and cortex and cerebellum showed a significant decrease. Functional magnetic resonance imaging (fMRI) are often used to decode stimuli, behaviors, and diseases through machine learning models [16]. Rixing Jing et al. [17] successfully differentiated patients with WDs from healthy controls (HCs) using machine learning classification based on the functional brain network of the disease. The study found that functional brain networks may serve as a biomarker to differentiate WDs from HCs. However, the lack of external validation calls for further investigation to assess the generalizability of these findings. Amplitude of low-frequency fluctuations (ALFF) based on rs-fMRI is an indicator used to measure the changes in resting blood oxygen level-dependent (BOLD) signals, which are often used to reflect brain activity [18]. There were significantly altered ALFF values of some brain regions in WDs compared with those of HCs [19,20]. In recent years, some research on ALFF-based classification has shown good performance. In Parkinson's patients, the treatment response to levodopa was successfully predicted through ALFF [21]. In patients with primary dysmenorrhea, the accuracy of distinguishing patients from HCs reached 96.36% [22]. In previous studies, Yutong Wu et al. [23] have shown that using amplitude of low-frequency fluctuations (ALFF) and local coherence as features in combination with support vector machine (SVM) models can effectively differentiate patients with white matter degeneration WDs from HCs. However, these studies lacked external validation and did not investigate or explore the standalone classification ability of ALFF or the necessity of external validation in this field. In the field of neuroimaging data classification, SVM is one of the commonly used classifiers due to its strong classification performance and interpretability in high-dimensional data [24]. However, besides SVM, there are other classifiers that can be used for neuroimaging data classification, including the logistic regression (LR) model and the random forest (RF) model. LR is a probability-based regression model that can be used for classification problems, especially in binary classification problems where it is widely applied. LR and SVM usually have comparable performance, but the former is more naturally applicable to situations with more than two classes [16]. RF is an ensemble learning method that can effectively handle high-dimensional data and complex data relationships. In neuroimaging data classification, the RF model can perform feature selection and combination to improve classification accuracy and stability [25]. Therefore, besides SVM, LR and RF models also have significant application value in neuroimaging data classification, and the appropriate classifier can be selected based on the specific research question and data feature. This study represents the first application of machine learning classification based on ALFF, using multiple classifiers in WDs. Our objective was to train machine learning models to differentiate between WDs and HCs. Specifically, we employed ALFF data derived from clusters identified in inter-group comparisons to assess their potential as diagnostic biomarkers for WD. To enhance the model's classification performance, we incorporated volumetric information, clinical variables, and imaging features.

## 2 Methods and materials

### 2.1 Patient selection

From February 2022 to April 2022, we recruited 37 WDs who were hospitalized at the Affiliated Hospital of the Institute of Neurology, Anhui University of Chinese Medicine, as well as 30 HCs. The inclusion criteria for WDs followed the diagnostic criteria in the “Guidelines for Diagnosis and Treatment of Hepatolenticular Degeneration (2022)” [26]. HCs were recruited from various sources, including family members of patients, hospital staff, students, and others. Participants were aged ≥18 years old and had no history of craniocerebral trauma, infection, or other relevant conditions. We evaluated the Unified Wilson's Disease Rating Scale (UWDRS) in WDs and divided them into neurological and non-neurological subgroups based on the scale's neurological function assessment. All participants provided written informed consent. This study was conducted in accordance with the Helsinki Declaration and approved by the Ethics Committee of the Affiliated Hospital of the Institute of Neurology, Anhui University of Chinese Medicine.

### 2.2 Sample size estimation

Based on the analysis results with a confidence level of 0.05 and a confidence interval of 0.1, we determined that the positive to negative sample ratio was approximately 1:1. Using the receiver operating characteristic (ROC) curve, we evaluated the effect size and obtained an area under curve (AUC) of 0.8, which led to a final sample size of 42. To ensure the reliability and generalizability of our study, we plan to partition the dataset into training and testing sets in a 7:3 ratio and recruit 60 or more participants.

### 2.3 MRI acquisition

MRI scanning was performed using a 1.5T magnetic resonance scanner (Siemens Medical Solutions, Erlangen, Germany). During the scan, each subject was instructed to keep their eyes closed and remain awake. T1-weighted structural images were acquired using a 3D magnetization-prepared rapid gradient-echo (MPRAGE) sequence, with the following parameters: field of view (FOV) read = 250 mm, echo time (TE) = 3.31 ms, repetition time (TR) = 2200 ms, voxel size = 1 mm × 1 mm × 1 mm, and no layer gap. An echo-planar imaging sequence was used to obtain the resting state functional image. The parameters were: FOV read = 162 mm, TE = 50 ms, TR = 4000 ms, voxel size = 3.4 mm × 3.4 mm × 3 mm, layer gap = 0.8 mm, volumes = 180. During the resting state scan, subjects were instructed to keep their eyes closed, remain awake, and not think about anything in particular. To assess potential signal abnormalities, such as T1, T2, and FLAIR changes, in the thalamus, lenticular nucleus, caudate nucleus, cerebellum, and frontal-parietal lobe, we also obtained routine T2 and FLAIR imaging data. The T2 image parameters were as follows: FOV = 230 × 208, TE = 101 ms, TR = 4670 ms, slice thickness = 5 mm. The FLAIR image parameters were as follows: FOV = 230 × 208, TE = 107 ms, TR = 7500 ms, slice thickness = 5 mm.

### 2.4 Preprocessing and ALFF analysis

We used CONN21b to preprocess the fMRI and T1 images with the default preprocessing pipeline [27]. It mainly included functional realignment and unwarp, slice-timing correction, outlier identification, direct segmentation and normalization, and functional smoothing (6 mm). The denoising pipeline mainly included linear trend removal and regression covariates (motion, white matter, cerebrospinal fluid Signal, scrubbing, and session). After that, we calculated the ALFF (0.01–0.08 Hz). The ALFF maps were then transformed into z values using Fisher's r-to-z transformation. In quality assessment of preprocessed fMRI images, we examine for low frequency drifts, cardiac-related signal fluctuations, and motion-related artifacts. If these issues are detected, they can be addressed by applying filtering techniques or using improved cardiac or respiratory signal correction methods. In addition, scrubbing tool can be utilized to identify motion-related artifacts and exclude corresponding time points from the analysis.

### 2.5 Segmentation of the thalamus and caudate nucleus

The segmentation of subcortical nucleus including the thalamus and caudate nucleus, and the calculation of their volume and total intracranial volume (TIV), were carried out by FreeSurfer7.2 (http://surfer.nmr.mgh.harvard.edu/). The segmentation quality was visually inspected by 2 radiologists.

### 2.6 Statistical analysis and machine learning

Chi-square tests were used for categorical variables, independent samples t-tests for normally distributed continuous variables and Mann–Whitney tests for non-normally distributed continuous variables. Two-sided, P < 0.05 was defined as significant.

The study randomly assigned participants to a training set and a test set using a 7:3 ratio. The two-sample *t*-test was used to compare the ALFF z-values between two groups, with age and sex as covariates, at the voxel level. A significance threshold of P < 0.001 was set for voxels, and a significance threshold of P < 0.05 after correction for Gaussian random field (GRF) was set for clusters, with a minimum cluster size of 30 voxels. “Cluster” refers to a collection of adjacent voxels in space that exhibit significant differences between groups.

We identified 4 clusters with significant differences between the ALFF z-values of WDs and HCs. These clusters were defined as regions of interest (ROIs) in our study. Subsequently, we trained three machine learning models, namely SVM, RF, and LR, using the ALFF z-values of all voxels within each ROI. We evaluated the performance of our models using 10-fold nested cross-validation in the training set. This approach allowed for the ideal hyper-parameter to be determined using the internal folds, while the outer layers were used to assess the model's effectiveness [28]. The study evaluated the generalization capability of the models, calculating sensitivity, specificity, accuracy, and AUC of each model to assess their diagnostic performances. AUC values close to 1 (AUC >0.8) were considered indicative of good classifier models.

**Fig. 1:**
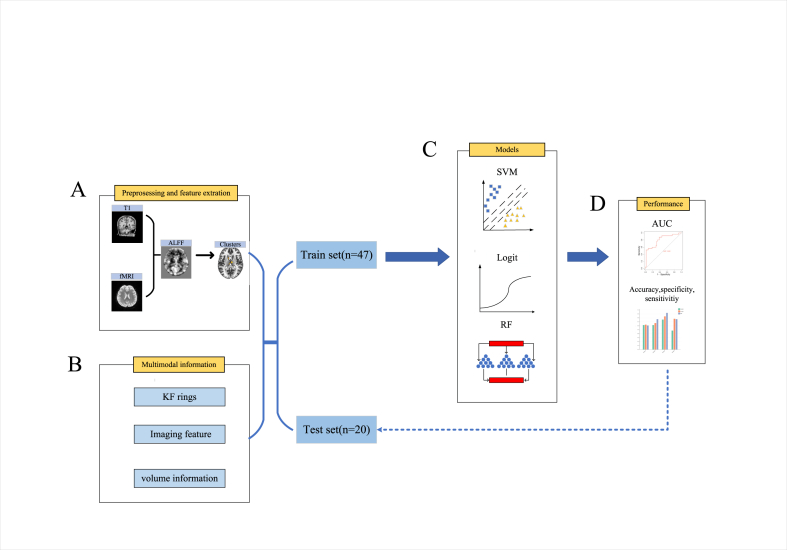
Machine Learning Classification Workflow for WDs and HCs. The flowchart consists of the following parts: Step A utilizes preprocessing of rs-fMRI to obtain the ALFF z-values in localized brain regions, followed by comparison of intergroup differences to obtain surviving clusters. B includes multimodal information consisting of neuroimaging features, clinical variables, and volumetric data. C involves three classifiers: SVM, LR, and RF. D comprises performance metrics for evaluating the model, including ROC curve, accuracy, sensitivity, and specificity.

After validation on the testing set, we found that models with AUC values greater than 0.8 were predominantly generated by training on clusters located in the thalamus or caudate regions, specifically Cluster 2, Cluster 3, and Cluster 4. Therefore, we used a linear regression model to evaluate differences in thalamus or caudate volume in the training set, while considering age, gender, and intracranial volume as covariates. Next, we transformed the volumes of the thalamus and caudate nuclei into z-scores, which we referred to as volume information. Then, we applied the same method to train and evaluate the model using the aforementioned clusters, as well as multimodal information consisting of clinical variables (KF ring), imaging features, and their corresponding volume information. We employed the Delong test to compare the AUC values among the models trained by these clusters before fusing the multimodal information, as well as to examine the differences in AUC values between the fused and non-fused models (Fig. 1). All statistical analyses were conducted using Python 3.9.

## 3 Results

### 3.1 Demographic and clinical variables

We recruited 67 subjects, including 37 WDs and 30 HCs. As shown in Table 1, the median ages of the HCs and WDs were 25 (24,27) and 27 (23,31) years old respectively (P = 0.246). The differences of gender between the two groups were statistically significant (P = 0.024). The differences of BMI (P = 0.837) and education years (P = 0.157) were not statistically significant. 23 WDs had neurological manifestations, and 24 WDs had KF rings. On T2-or FLAIR-weighted head MRI, 25 WDs exhibited abnormal signals in the thalamus, caudate nucleus, or lenticular nucleus, while 14 WDs exhibited abnormal signals in the midbrain/brainstem and 14 had abnormal signals in the frontal/parietal regions. There were no significant inter-group differences in KF rings among neurological and non-neurological WDs (P = 0.078). As indicated in Table 2, there were no statistically significant differences in age (P = 0.435) or disease duration (P = 0.343) between the training and testing sets of WDs.

**Table 1:** Demographic and clinical variables of HCs and WDs.

Variable	HC, N = 30	WD, N = 37	p value
**Age (years)**	25 (24,27)	27 (23,31)	0.246
**Gender (Y/A)**			0.024
**Female**	22 (73%)	17 (46%)	
**Male**	8 (27%)	20 (54%)	
**Education years (years)**	11.5 (9,15.5)	10 (9,12)	0.157
**BMI (kg/m**^**2**^**)**	21.13 ± 2.54	21.29 ± 3.34	0.837
**WD type (neurological/non-neurological)**	–	23/14	–
**Disease duration (years)**	–	12.49 ± 7.57	–
**KF rings (Y/A)**	0/30	26/37	0.000
**Abnormal MRI signals (Y/A)**			
**Lenticular nucleus/thalamus/caudate**	0/30	25/37	0.000
**Cerebellum/brainstem**	0/30	14/37	0.000
**Frontoparietal**	0/30	14/37	0.000

Variables conforming to the normality test are represented by the mean ± sd, while variables not conforming to the normality test are represented by the median (IQR). (Y/A) refers to Yes/all.

**Table 2:** Comparison of demographics among WDs in the test and Training sets.

Variable	test set (n = 11)	train set (n = 26)	p value
**Age (years)**	26.27 ± 6.62	28.31 ± 7.12	0.435
**Gender (Y/A)**			0.160
**Female**	7 (63.636)	10 (38.462)	
**Male**	4 (36.364)	16 (61.538)	
**Disease duration (years)**	8.00 (3.00,20.00)	13.00 (8.00,20.00)	0.343

Variables conforming to the normality test are represented by the mean ± sd, while variables not conforming to the normality test are represented by the median (IQR). (Y/A) refers to Yes/all.

### 3.2 Intergroup differences in the comparison of ALFF-z values between the WDs and the HCs

**Fig. 2:**
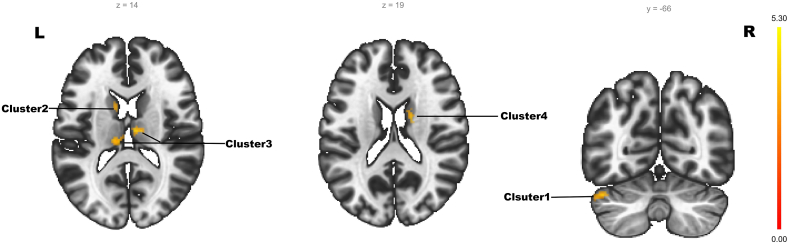
There were 4 Clusters with increased ALFF z-values compared with HCs. Cluster1 is in the cerebellum region, Cluster2 was mainly in the left caudate nucleus, Cluster3 was in the bilateral thalamus, and Cluster4 was in the right caudate nucleus. In MNI space, the y-axis represents the distance in the forward direction (negative for backward) from the origin, and the z-axis represents the distance in the upward direction (negative for downward) from the origin. L/R denotes the left/right hemisphere.

As shown in Table 3, in the training set, there were 4 survived clusters which showed significantly altered ALFF z-values in the WDs compared with HCs. Cluster1 was in the cerebellar region, Cluster2 was mainly in the left caudate nucleus, Cluster3 was in the bilateral thalamus, and Cluster4 was in the right caudate nucleus (Fig. 2).

**Table 3:** Significant clusters with different ALFF z-values of intergroup comparisons.

			Brain areas	MNI peak (x, y, z)	Cluster size (voxels)	Peak T
WDs > HCs						
**Cluster1**			L-cerebellum	−44, −66, −28	86	4.1109
**Cluster2**			l-caudate	−14, +18, +4	110	4.5006
**Cluster3**			L-thalamus	+10, −14, +12	202	5.3001
			R-thalamus			
**Cluster4**			R-caudate	+2, −68, +50	76	4.7595

L/R represents left/right brain regions. The MNI peak refers to the MNI space coordinates of the peak in the Cluster.

### 3.3 Performance of the models based on ALFF in the training set

**Fig. 3:**
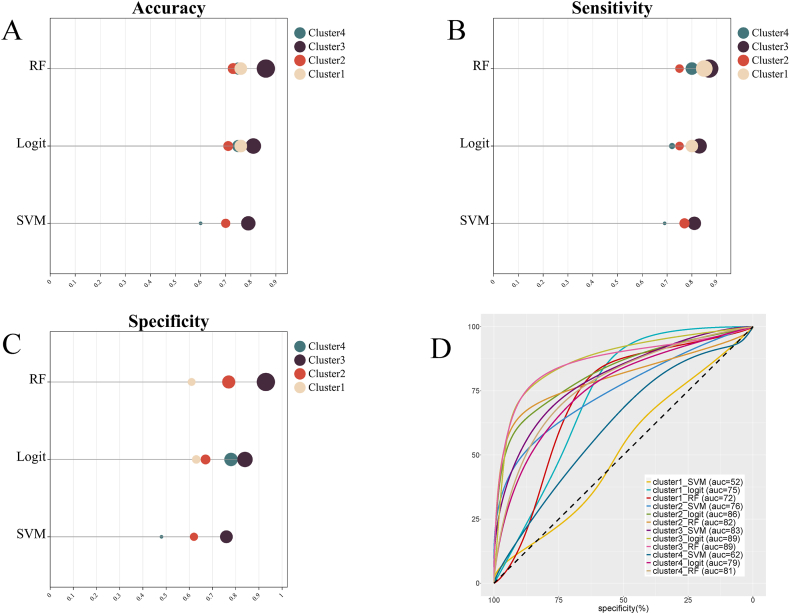
Classification performance of each model in the training set. (A) Refers to accuracy, (B) refers to sensitivity, (C) refers to specificity, (D) refers to AUC, and the unit is the percentage. The curve is smoothed.

Table 4 shows the classification performance of all classification models based on ALFF-z values. In the training, some models trained with Cluster2, Cluster3, and Cluster4 achieved AUC greater than 0.80. Trained with Cluster2, the AUC of the LR model (AUC = 0.86, accuracy = 0.71, sensitivity = 0.75, specificity = 0.67) and RF model (AUC = 0.82, accuracy = 0.73, sensitivity = 0.75, specificity = 0.77) exceeded 0.80. Trained with Cluster3, the AUC of the SVM model (AUC = 0.83, accuracy = 0.79, sensitivity = 0.81, specificity = 0.76), LR model (AUC = 0.89, accuracy = 0.81, sensitivity = 0.83, specificity = 0.84), and RF model (AUC = 0.89, accuracy = 0.86, sensitivity = 0.87, specificity = 0.93) all exceeded 0.80. Trained with Cluster4, the AUC of the RF model (AUC = 0.81, accuracy = 0.75, sensitivity = 0.80, specificity = 0.77) exceeded 0.8 (Fig. 3).

**Table 4:** Classification performance of the models based on ALFF.

Classification model	accuracy	sensitivity	specificity	AUC (95% CI)
**Training sets**				
**Cluster1**				
SVM	0.71	0.82	0.62	0.52 (0.37, 0.67)
LR	0.76	0.80	0.63	0.75 (0.60, 0.87)
RF	0.76	0.85	0.61	0.72 (0.57, 0.84)
**Cluster2**				
SVM	0.70	0.77	0.62	0.76 (0.62, 0.88)
LR	0.71	0.75	0.67	**0.86 (0.72, 0.94)**
RF	0.73	0.75	0.77	**0.82 (0.68, 0.91)**
**Cluster3**				
SVM	0.79	0.81	0.76	**0.83 (0.69, 0.92)**
LR	0.81	0.83	0.84	**0.89 (0.77, 0.97)**
RF	0.86	0.87	0.93	**0.89 (0.76, 0.96)**
**Cluster4**				
SVM	0.60	0.69	0.48	0.62 (0.47, 0.76)
LR	0.75	0.72	0.78	0.79 (0.65, 0.90)
RF	0.75	0.80	0.77	**0.81 (0.67, 0.91)**
**Test sets**				
**Cluster1**				
SVM	0.60	0.55	0.61	0.61 (0.37, 0.81)
LR	0.60	0.55	0.67	0.65 (0.41, 0.85)
RF	0.65	0.45	0.89	0.67 (0.42, 0.86)
**Cluster2**				
SVM	0.65	1.00	0.22	**0.94 (**0.74, 1.00)
LR	0.75	1.00	0.44	**0.94 (0.72, 1.00)**
RF	0.65	0.91	0.33	**0.91 (0.70, 0.99)**
**Cluster3**				
SVM	0.70	0.82	0.66	0.76 (0.52, 0.92)
LR	0.80	0.82	0.78	**0.84 (0.61, 0.97)**
RF	0.82	0.82	0.78	**0.87 (0.64, 0.98)**
**Cluster4**				
SVM	0.75	1.00	0.44	**0.87 (0.65, 0.98)**
LR	0.70	1.00	0.33	**0.93 (0.72, 1.00)**
RF	0.70	1.00	0.33	**0.90 (0.67, 0.91)**

The performance of the models was examined for accuracy, specificity, sensitivity, and AUC. CI refers to confidence interval.

### 3.4 External validation in the test set

As shown in Table 4, trained with Cluster2, the AUC of the SVM model (AUC = 0.94, accuracy = 0.65, sensitivity = 1.00, specificity = 0.22) and LR model (AUC = 0.94, accuracy = 0.75, sensitivity = 1.00, specificity = 0.44), and RF model (AUC = 0.91, accuracy = 0.65, sensitivity = 0.91, specificity = 0.33) all exceeded 0.80. Trained with Cluster3, the AUC of the LR model (AUC = 0.84, accuracy = 0.80, sensitivity = 0.82, specificity = 0.78) and RF model (AUC = 0.87, accuracy = 0.82, sensitivity = 0.82, specificity = 0.78) exceeded 0.80. Trained with Cluster4, the AUC of the SVM model (AUC = 0.87, accuracy = 0.75, sensitivity = 1.00, specificity = 0.44), LR model (AUC = 0.93, accuracy = 0.70, sensitivity = 1.00, specificity = 0.33), and RF model (AUC = 0.90, accuracy = 0.70, sensitivity = 1.00, specificity = 0.33) all exceeded 0.80. As shown in Table 5, statistical significance was observed only in the AUC values of models trained with Cluster4 in the Delong test. Specifically, both the Logit model (P = 0.04) and RF model (P = 0.04) demonstrated significantly higher AUC values compared to the SVM model.

**Table 5:** Comparison of AUC values among models in the testing set before integration of multimodal information.

	cluster2-SVM	cluster2-Logit	cluster2-RF	cluster3-SVM	cluster3-Logit	cluster3-RF	cluster4-SVM	cluster4-Logit	cluster4-RF
**cluster2-SVM**	1.000	1.000	0.599	0.563	0.941	0.866	0.777	0.206	0.330
**cluster2-Logit**	1.000	1.000	0.507	0.539	0.939	0.861	0.767	0.225	0.279
**cluster2-RF**	0.599	0.507	1.000	0.330	0.658	0.827	0.455	0.464	0.542
**cluster3-SVM**	0.563	0.539	0.330	1.000	0.194	0.172	0.813	0.141	0.171
**cluster3-Logit**	0.941	0.939	0.658	0.194	1.000	0.557	0.856	0.296	0.353
**cluster3-RF**	0.866	0.861	0.827	0.172	0.557	1.000	0.652	0.384	0.440
**cluster4-SVM**	0.777	0.767	0.455	0.813	0.856	0.652	1.000	0.050	**0.043**
**cluster4-Logit**	0.206	0.225	0.464	0.141	0.296	0.384	0.050	1.000	0.782
**cluster4-RF**	0.330	0.279	0.542	0.171	0.353	0.440	**0.043**	0.782	1.000

Pairwise comparison of AUC values using the DeLong test. The values in the table indicate the P values for the comparison between the corresponding row and column models. These models refer to the SVM, RF, and LR models trained by cluster2, cluster3, and cluster4, respectively.

**Fig. 4:**
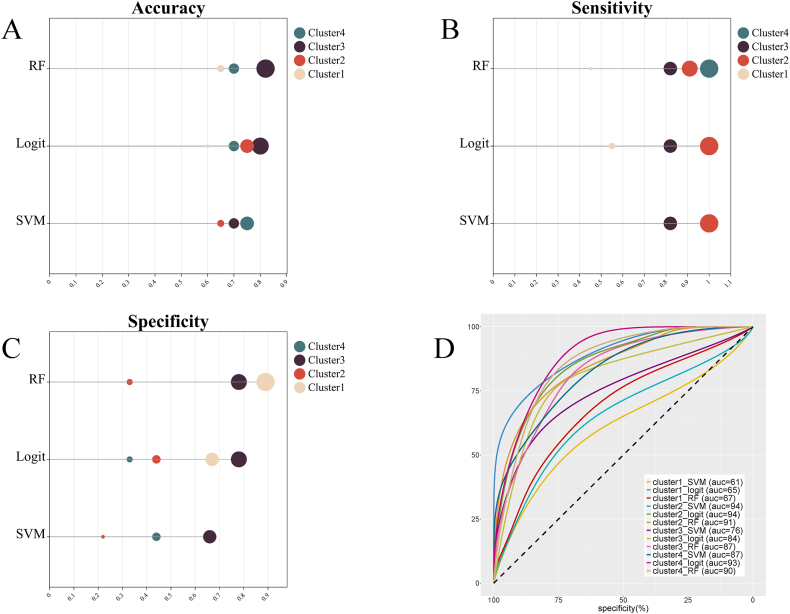
Classification performance of each model in the test set. (A) Refers to accuracy, (B) refers to sensitivity, (C) refers to specificity, (D) refers to AUC, and the unit is the percentage. The curve is smoothed.

In summary, in the test set, some classification models trained with Cluster 2, Cluster 3, and Cluster 4 had good classification performance. Among these models, the models trained with Cluster 2 or Cluster 4 showed high sensitivity, low specificity, and low accuracy. The LR model and RF model trained with Cluster 3 showed high specificity, sensitivity, and accuracy (Fig. 4).

### 3.5 Volume differences of the caudate nucleus and thalamus

**Fig. 5:**
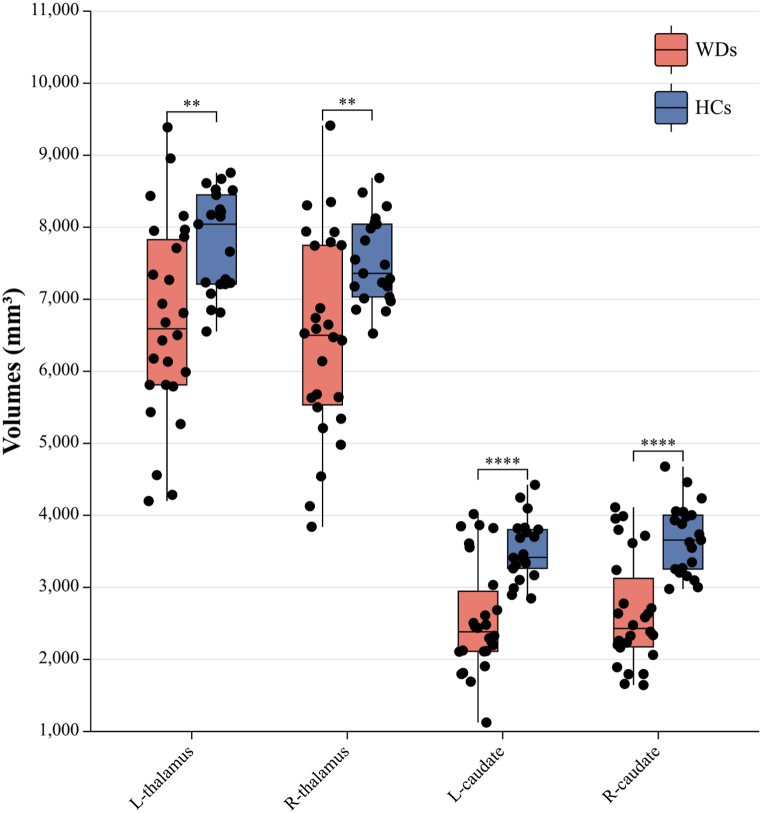
Differences in the volumes of the bilateral thalamus and caudate nucleus in the training set. The volumes of the bilateral thalamus and caudate nucleus in WDs were atrophied compared with those in HCs. Unit:mm³.

As shown in Table 6, in the training set, the volumes of the thalamus (L, P = 0.005; R, P = 0.004) and caudate nucleus (L, P = 0.000; R, P = 0.000) on both sides of the WDs were significantly smaller than those of the HCs (Fig. 5).

**Table 6:** Differences in the volumes of the bilateral thalamus and caudate nucleus in the training set.

Variables	HC, N = 21	WD, N = 26	p value
**L-thalamus**	7783.514 ± 708.849	6686.396 ± 1381.869	0.005
**R-thalamus**	7522.267 ± 600.008	6467.069 ± 1405.744	0.004
**l****-caudate nucleus**	3520.995 ± 428.351	2568.400 ± 773.927	0.000
**R-caudate nucleus**	3673.776 ± 488.138	2653.912 ± 769.850	0.000

L/R represents left/right brain regions. All variables are expressed as the mean ± sd. All variables are in mm^3^.

### 3.6 Classification performance of models with integrated multimodal information in the testing set

**Fig. 6:**
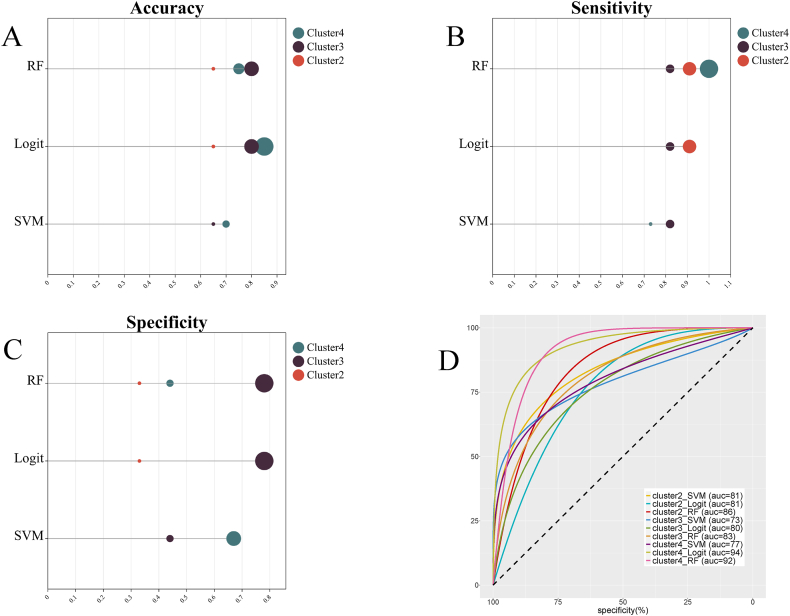
Performance in the test set of the models trained with ALFF z-values, volume information, clinical variables, and imaging features. (A) Refers to accuracy, (B) refers to sensitivity, (C) refers to specificity, (D) refers to AUC, and the unit is the percentage. The curve is smoothed.

Table 7 displays the performance evaluation results of the models trained with clusters, including multimodal information comprising volume information, clinical features, and imaging features, in the test set. For the models trained with Cluster2 and multimodal information, the SVM model (AUC = 0.81, accuracy = 0.65, sensitivity = 1.00, specificity = 0.22), LR model (AUC = 0.65, accuracy = 0.91, sensitivity = 0.33, specificity = 0.33), and RF model (AUC = 0.86, accuracy = 0.65, sensitivity = 0.91, specificity = 0.33) all exceeded an AUC value of 0.80. Similarly, for the models trained with Cluster3 and multimodal information, the AUC of the LR model (AUC = 0.8, accuracy = 0.80, sensitivity = 0.82, specificity = 0.78) and RF model (AUC = 0.83, accuracy = 0.8, sensitivity = 0.82, specificity = 0.78) both exceeded 0.80. For the models trained with Cluster4 and multimodal information, the AUC of the LR model (AUC = 0.94, accuracy = 0.85, sensitivity = 0.91, specificity = 0.78) and RF model (AUC = 0.92, accuracy = 0.75, sensitivity = 1.00, specificity = 0.44) both exceeded 0.80 (Fig. 6). As shown in Table 8, there was no statistically significant difference in the AUC values between the model incorporating multimodal information and the model without such integration. The LR models trained with multimodal information and Cluster4, as well as the LR and RF models trained with multimodal information and Cluster3, showed high accuracy, specificity, and sensitivity.

**Table 7:** Performance in the test set of the models trained with ALFF z-values, volume information, clinical features.

Classification model	Accuracy	sensitivity	specificity	AUC (95%CI)
**Cluster2**				
SVM	0.65	1.00	0.22	**0.81 (0.57,0.95)**
LR	0.65	0.91	0.33	**0.81 (0.57,0.97)**
RF	0.65	0.91	0.33	**0.86 (0.63,0.97)**
**Cluster3**				
SVM	0.65	0.82	0.44	0.73 (0.49,0.90)
LR	0.80	0.82	0.78	**0.80 (0.56,0.94)**
RF	0.80	0.82	0.78	**0.83 (0.60,0.96)**
**Cluster4**				
SVM	0.70	0.73	0.67	0.77 (0.53,0.92)
LR	0.85	0.91	0.78	**0.94 (0.73,0.99)**
RF	0.75	1.00	0.44	**0.92 (0.71,0.99)**

The performance of the models was examined for accuracy, specificity, sensitivity, and AUC on the test set. CI refers to confidence interval.

**Table 8:** Comparison of AUC values among models before and after integration of multimodal information in the testing set.

	Classifier	P
**Cluster2**	**SVM**	0.378
	**LR**	0.164
	**RF**	0.209
**Cluster3**	**SVM**	0.246
	**LR**	0.109
	**RF**	0.540
**Clsuter4**	**SVM**	0.269
	**LR**	1.000
	**RF**	1.000

Using the DeLong test, we compared the AUC values between models before and after integration of multimodal information.

## 4 Discussion

In this study, we obtained four Clusters with abnormal ALFF z-values in the intergroup comparison. Cluster1 was in the cerebellum region, Cluster2 was mainly in the left caudate nucleus, Cluster3 was in the bilateral thalamus, and Cluster4 was in the right caudate nucleus. Some model trained with Cluster2, Cluster3, and Cluster4 showed good discrimination ability in both training and test sets. In the Delong test, only the LR and RF models trained with Cluster4 demonstrated higher AUC values compared to the SVM model. Additionally, the LR and RF models trained with Cluster3 exhibited exceptional sensitivity, specificity, and accuracy on the test set. Then, we trained the model with multimodal information included ALFF z-values, volume information, clinical variables, and imaging features and evaluated the model performance in the test set. There was no statistically significant improvement in AUC values for each model that incorporated multimodal information compared to their previous versions. The LR models trained with multimodal information and Cluster4, as well as the LR and RF models trained with multimodal information and Cluster3, showed high accuracy, specificity, and sensitivity. In the present study, compared with the traditional approach of employing a single support vector machine (SVM) model for classifying all ALFF abnormalities, we propose a cluster-based classification strategy. We first conduct between-group comparisons to identify distinct clusters, and then train multiple classifiers for each cluster. Modeling each cluster separately allows for better discrimination of these brain regions, thereby facilitating the uncovering of underlying neurophysiological mechanisms. The novelty of utilizing different classifiers lies in the enhanced ability to mine the potential information within each cluster's features, leveraging the strengths of various classifiers to improve overall classification performance. This advantage is expected to provide a more accurate basis for diagnosis and prognosis assessment in clinical applications. External validation was conducted in this study, offering a more objective evaluation of the model's generalization capability.

Cluster 2, 3, and 4 were located in the bilateral thalamus and right/left caudate nucleus, respectively. The thalamus is an oval gray matter nucleus located in the diencephalon on both sides of the third ventricle. The thalamus is the central sensory and motor relay station of the brain and plays a key role in primary sensory processing, sleep, arousal, and cognitive processes [29]. The caudate nucleus is located near the lateral ventricle and is a component of the striatum. Both are important components of the basal ganglia thalamus cortex circuit and jointly regulate the motor function of the body [30]. Studies have found that the thalamic lesions are related to depression, and the caudate nucleus lesions are associated with anxiety [31]. Consistent with our study, several previous studies have confirmed that the volume atrophy of the bilateral caudate nucleus and thalamus in WDs is higher than that in HCs [10,32,33]. Diffusion tensor-weighted imaging shows an increase in fractional Anisotropy (FA) in both the thalamus and caudate nucleus, with a close relationship between FA in the bilateral thalamus and cognition, according to several studies [[34], [35], [36]]. Previous studies also found that the ALFF z-values of the basal ganglia and thalamus in WDs were higher than those in HCs [19,20]. On quantitative magnetic susceptibility imaging, it was found that the brain iron content of the subcortical nuclei, including the thalamus and caudate nucleus, in WDs was higher than that in the control group [10,37,38]. These results indicate that there is functional or organic damage in the thalamus and caudate nucleus in WDs. The accumulation of copper in the brain in WD may contribute to pathological changes such as myelin sheath degeneration, astrocyte proliferation, and severe neuron loss, as suggested by previous studies [39]. These studies suggest that the thalamus and caudate nucleus play important roles in the neuropathological basis of WD, which supports our findings of good performance of machine learning models.

In the test set, we found that the models trained with clusters located in the caudate nucleus showed low accuracy and low specificity. This may be related to the clinical heterogeneity of WDs. WDs often have different clinical manifestations due to variable levels of copper accumulation throughout their body. This may be why the classification models showed low specificity and low accuracy. The sensitivity, specificity, accuracy, and AUC of the LR model and RF model trained with Cluster3 showed higher. This may be related not only to the copper damage of WDs but also to liver damage. Several studies have found that nonalcoholic fatty liver disease, cirrhosis, hepatic encephalopathy, and other liver diseases are closely related to the impairment of thalamic function [[40], [41], [42], [43]]. The reason why some models trained with Cluster 3 showed high AUC, accuracy, specificity, and sensitivity may be due to the fact that liver damage in WDs also affects the function of the thalamus.

Almost all the Logit and RF models trained on clusters had higher AUC values than the SVM model, and the Logit and RF models trained on cluster 4 had significantly higher AUC values than the SVM model. The reasons behind this may be as follows: Firstly, in the case of small samples with multiple features, SVM may be too complex and prone to overfitting, while Logistic regression and random forest models may perform better [44]. Secondly, SVM requires the appropriate kernel function and regularization parameter selection, while Logistic regression and random forest models are relatively simple and do not require extensive parameter selection and tuning. Finally, the impact of noise and outliers may also contribute to the difference in performance. SVM is sensitive to noise and outliers and can be affected by these factors, whereas Logistic regression and random forest models are relatively robust to such factors [22]. At present, the diagnosis of WD is based on gene mutation detection, serum ceruloplasmin, urine copper determination, KF ring, liver copper, liver histology, and imaging features [45,46]. However, the application of rs-fMRI is rare. It reported that the SVM model based on functional brain network training showed a good ability to distinguish between WDs and HCs [17]. However, there is a lack of research on the recognition of WDs based on ALFF. This study is the first classification study based on ALFF. Some models trained with clusters located in the caudate nucleus and thalamus had good performance. This means that the diagnosis of WDs based on ALFF is feasible.

## 5 Conclusions

This study demonstrated high classification performance in distinguishing WDs from those with HCs. ALFF based on the thalamus or caudate nucleus has the potential to serve as a biomarker for distinguishing between patients with WDs and HCs. The fusion of multimodal information, including imaging features, volume information, and clinical variables, apparently did not significantly improve the classification performance of the model.

However, there are several limitations that should be addressed. Firstly, machine learning models such as SVM, RF, and LR have inherent limitations that may affect their performance and effectiveness in certain situations. For example, the Logit model cannot solve collinearity problems and is sensitive to multicollinearity data. The SVM model has difficulty finding an appropriate kernel function and processing nonlinear problems. The RF model struggles with processing high-dimensional data and has poor interpretability. Therefore, techniques such as parameter tuning, feature selection and transformation, and regularization are necessary to overcome these limitations. Secondly, although this study did not find any statistically significant gender differences, there was an imbalance in the data. This imbalance has the potential to introduce bias and subsequently affect the performance of the models. Future studies will aim to balance the number of male and female samples during the recruitment and data analysis stages. Thirdly, the subtypes of WD were not classified due to the small sample size of this study. In a follow-up study, we will recruit more subjects to distinguish the subtypes of WD and HCs. Finally, the limited image quality obtained from MRI scans acquired with a 1.5T scanner is a limitation of this study. In the future, image acquisition will be performed on a 3.0T scanner. In addition, future studies will integrate multiple modalities of information such as local consistency, functional connectivity matrix, and employ machine learning techniques to identify distinct subtypes of WD.

In conclusion, while this study demonstrates promising results, there is still room for improvement. Addressing the limitations identified in this study will enable the development of more accurate and reliable classification models for WD diagnosis.

## Funding

This research was funded by Anhui University of Chinese Medicine, China grant num-ber2021sfylc01.

## Author contribution statement

Bing Zhang: Conceived and designed the experiments; Performed the experiments; Analyzed and interpreted the data; Wrote the paper. Jingjing Peng, Hong Chen: Performed the experiments; Analyzed and interpreted the data. Wenbin Hu: Contributed reagents, materials, analysis tools or data; Wrote the paper.

## Data availability statement

Due to the requirements of the affiliated institution, the original data have not been deposited in a public database. However, in the interest of research integrity and transparency, we assure that the corresponding author can provide the relevant data upon request.

## Additional information

The study was conducted in accordance with the Declaration of,.Helsinki, and approved by the Ethics Committee of the Affiliated Hospital of the Institute of Neurology, Anhui University of Chinese Medicine (Ethics Approval No. 2021-Lun-Zi (13), translated from Chinese).

## Declaration of competing interest

The authors declare that they have no known competing financial interests or personal relationships that could have appeared to influence the work reported in this paper.
